# VEZT, a Novel Putative Tumor Suppressor, Suppresses the Growth and Tumorigenicity of Gastric Cancer

**DOI:** 10.1371/journal.pone.0074409

**Published:** 2013-09-17

**Authors:** Ruizhen Miao, Xiaobo Guo, Qiaoming Zhi, Yulong Shi, Leping Li, Xuehui Mao, Li Zhang, Chensheng Li

**Affiliations:** 1 Department of Gastrointestinal Surgery, Provincial Hospital Affiliated to Shandong University, Jinan, Shandong, China; 2 Department of General Surgery, the First Affiliated Hospital of Soochow University, Suzhou, Jiangsu, China; National Cancer Center, Japan

## Abstract

Vezatin (VEZT), an adherens junctions transmembrane protein, was identified as a putative tumor suppressor in our previous study. However, the role of VEZT in tumorigenesis remains elusive. We aimed to clarify its epigenetic regulation and biological functions in gastric cancer. In this study, we show that the expression level of VEZT is involved in lymphatic metastasis, depth of cancer invasion and TNM stage in 104 gastric cancer patients. Bisulfate sequencing polymerase chain reaction (BSP) methods showed that VEZT was hypermethylated in tissues and corresponding blood of gastric cancer patients compared with healthy controls. *Helicobacter pylori* (*H. pylori*) infection induces the methylation and silencing of VEZT in GES-1 cells. Restoring VEZT expression in MKN-45 and NCI-N87 gastric cancer cells inhibited growth, invasion and tumorigenesis *in vitro and in vivo*. Global microarray analysis was applied to analyze the molecular basis of the biological functions of VEZT after VEZT transfection combined with real-time PCR and chromatin immunoprecipitation assay. G protein-coupled receptor 56(GPR56), cell growth, cell division cycle 42(CDC42), migration/invasion and transcription factor 19(TCF19), cell cycle progression, were identified as direct VEZT target genes. TCF19, a novel target of VEZT, was functionally validated. Overexpression of TCF19 in MKN-45 cells increased cell cycle progress and growth ability. This study provides novel insight into the regulation of the VEZT gene, which could represent a potential target for therapeutic anti-cancer strategies.

## Introduction

Gastric cancer is the second leading cause of male cancer-related death and the third leading cause for female cancer-related death worldwide [1]. It is a major public health problem throughout the world [2]. In China, there are 400,000 new cases of gastric cancer and 300,000 deaths annually. Many cases who suffered from gastric cancer have lost curative chance with extremely poor outcome [3]. Therefore, developing novel and effective therapeutic methods is essential to reduce gastric cancer mortality. It has been known that the pathogenesis of gastric carcinomas is multifactorial, which includes genetic predisposition and environmental factors. There are a number of genetic alternations including tumor suppressor genes, oncogenes, cell adhesion molecules and growth factors [4].

Although the molecular mechanisms of gastric carcinogenesis remain unclear, epigenetic silencing of tumor-related genes by promoter hypermethylation has recently emerged as an important mechanism of tumorigenesis. The promoter hypermethylation profile differs in each cancer type and within each gene, providing tumor type- and gene-specific hypermethylation profiles that may involve in the corresponding molecular mechanism of tumorigenesis. The identification of a novel gene targeted by promoter hypermethylation may provide insights into the mechanisms for the inactivation of the tumor-suppressive pathways and is important for the identification of tumor markers in gastric cancer [5,6].

Recently, we have identified, an adherens junctions transmembrane protein, VEZT as a candidate tumor-suppressor gene. We aimed to clarify the epigenetic regulation and biological functions of VEZT in gastric cancer. VEZT, a ubiquitous integral protein, is indirectly associated with E-cadherin-catenin complex and actin cytoskeleton [7,8]. Its functions have mainly been explored in epithelial cells. Loss of VEZT is progressively detrimental to embryonic development [9,10], and VEZT is critical for preserving the integrity of the cellular junctions during long-term mechanical stress occurring at the level of inner ear hair cells in response to sound. Moreover, the inner ear-specific VEZT conditional invalidation leads to progressive deafness [7]. VEZT is highly expressed in the brain [8,11]. VEZT is enriched in dendritic spines in mouse hippocampal neurons. Using VEZT knock-down and conditional knockout before (D6cre) or after (CamKIIαcre) birth, VEZT regulates spine morphology. Morphological changes are not associated with compromised synaptic contacts, but postsynaptic VEZT plays a critical role in the morpho-functional maturation of excitatory postsynaptic elements [12].

Inactivation of the VEZT gene has been identified in gastric cancer, and the methylation of CpG islands within the promoter region of the VEZT gene contribute to its inactivation as determined by a previous study conducted by our lab [13]. The mechanism of the methylation and the exact role of VEZT in the development of gastric cancer are currently unknown. The target genes and related pathways of VEZT have not yet been identified. In this study, we systematically analyzed the mechanism of methylation and methylation status of the promoter of the VEZT gene. We also analyzed its functional features after reconstitution of VEZT expression *in vitro and in vivo*.

## Materials and Methods

### Cell lines and tissue samples

MKN-45, MKN-28, SGC-7901, the immortalized human gastric mucosal cell line GES-1(provided by the institute of digestive surgery of Ruijin hospital affiliated to Shanghai Jiao Tong University) [13,14,15,16,17] and human umbilical vein endothelial cells (HUVECs) were preserved in our institute. Gastric cancer cell lines SNU-1 and NCI-N87 cells were obtained from American Type Culture Collection (Manassas, VA, USA). Briefly, cells were grown in RPMI1640 supplemented with 10% fetal calf serum and 2 mM L-glutamine. Cells were maintained at 37°C in the presence of 5% CO_2_.

Primary gastric tumor and normal gastric mucosal tissues were collected from either routine therapeutic surgery or gastrointestinal endoscopy at our department. The remainder was *H. pylori* infection status was determined based on the rapid urease test as previously described [18].

### Ethics Statement

Written informed consent in the study was obtained from all participants. 4-week-old male BALB/c nude mice were purchased from the Experimental Animal Center of Chinese Academy of Sciences (Shanghai, China) and maintained in the Animal Laboratory Center of the Provincial Hospital Affiliated to Shandong University (Jinan, China) on a 12/12 h light/dark cycle (lights off at 19: 00) with food and water available adlibitum. The animal experiments were approved by the Institutional Animal Care and Use Committee at the Provincial Hospital Affiliated to Shandong University (Permit Number: SHANS87492). The study protocol was approved by the ethics committee of the Provincial Hospital Affiliated to Shandong University.

### Processing of Laser microdissection for tissues and cells

Tissues were removed as soon as possible after resection and fixed in formalin, embedded in paraffin, and cut into 8-µm-thick sections for hematoxylin and eosin (H&E) staining. All tissues were examined histologically, and experienced pathologists confirmed the diagnoses. A part of each sample was embedded in Tissue-Tek^®^ Optimum Cutting Temperature™ (OCT) compound medium (VWR Scientific Products, San Diego, CA, USA) in a cryostat and snaps frozen for microdissection.

Cells and frozen section slides were stained just before laser capture microdissection (LCM) on ice. Briefly, the sections were laser microdissected using a LM200 system (Olympus, Japan/Arcturus Engineering Inc, US). Areas of interest were selected under microscopic guidance, and covered with ethylene vinyl thermoplastic (EVA) film mounted on optically transparent cap. The infrared laser was activated by the push of a button, which melts the film directly above the target cells. This melt caused a binding to form between the cells and the transfer film that was stronger than the binding between the cells and the slides. The parameters used for LCM included a laser diameter of 7.5 µm, laser power of 50-60 mW. Five thousand laser pulse discharges per specimen were used to "capture" approximately 10 000 morphologically cells from each case. Each population was estimated to be >95% "homogeneous" as determined by microscopic visualization of the captured cells. The caps with captured cells were then fitted onto 0.5 mL microcentrifage tubes. After microdissection, the DNA, RNA, or protein can be extracted from aliquots of microdissected samples.

### Methylation analysis

Genomic DNA obtained from the microdissected cell lines, gastric cancer tissues and plasma (0.2 ml) was purified using DNAzol (Invitrogen). Purified DNA was treated with sodium bisulfite (Sigma, Phoenix, USA) and then analyzed by BSP or specific polymerase chain reaction (MSP) as previously described [13,15]. Amplified bisulfite PCR products were subcloned into a TA vector system (Promega) according to the manufacturer’s instructions. DNA sequencing was performed on three individual clones (Sangong). The PCR products were confirmed by agarose gel electrophoresis and visualized using ethidium bromide staining. The primers used are summarized in Table S1.

### Electron microscopic observation

*H. pylori* strains NCTC11637 (both CagA- and VacApositive) were provided by Professor Guo of the Department of Medical Microbiology and Parasitology, Institutes of Medical Sciences, Shanghai Jiao Tong University, School of Medicine. *H. pylori* strains were cultured routinely for 72 h on Columbia agar base (bioMérieux, France) with 5% sheep blood in mixed air containing 10% CO_2_, 5% O2, and 85% N_2_ at 37°C. Then, we converted *H. pylori* to liquid medium containing brain heart infusion (BD, U.S.), 10% sheep blood, and the same antibiotics as those used in Columbia agar base. The liquid medium was shaken on a shaker (Forma Scientific, U.S.) with a constant rotation rate of 120 rpm. *H. pylori* were counted using a spectrophotometer (BioSpec-min, Shimadzu Scientific Instruments, Japan) and washed with sterile PBS (pH 7.4, 5000 rpm, 10 min) before use. GES-1 cells (4 × 10^5^) were grown until confluent on glass cover slips in six-well plates, and then GES-1 cells were infected with *H. pylori* at an Multiplicity of infection (MOI) of 100:1. After incubation for 24 h, the morphological changes of GES-1 cells were observed by using an H-800 transmission electron microscope.

### Real-time qRT-PCR analysis

Purified total RNA was obtained from the microdissected cells, total RNA was extracted using Trizol solution. Reverse transcription (RT) was performed in a 20-µL reaction according to the manufacturer’s recommendations (Qiagen). Real-time qRT-PCR analyses were performed using primers listed in Table S1. Transcript expression levels were determined by quantifying the intensity of the PCR product normalized to glyceraldehyde-3-phosphate dehydrogenase (GAPDH) expression. Quantitative measurement of mRNA levels was performed using the ABI Prism 7000 (Applied Biosystems, Foster City, USA). These data were analyzed by using the comparative Ct method.

### Western blotting

Total protein was extracted from the microdissected cells. Signal protein extraction buffer 1 system (430-7608-MSDS) of Bio-Rad Corporation was used for protein extraction in our experiments and concentration was measured by the DC protein-assay method of Bradford (Bio-Rad). A total of 100 µg of protein from each sample were separated by 10% SDS-PAGE gel and transferred to an equilibrated polyvinylidene difluoride membrane (Amersham Biosciences, Buckinghamshire, UK) The proteins were detected by enhanced chemiluminescence (Amersham Corporation, Arlington Heights, IL, USA) after incubation with specific primary antibody VEZT(1:2,000), IL-6(1:2,000), AKT(1:1,000), Cag A(1:3,000), IL-8(1:3,000), ATF3(1:2,000), and IRX5(1:2,000) at 4°C overnight and then the secondary antibody. Protein levels were normalized to total GAPDH using a monoclonal anti-GAPDH antibody (Sigma) as previously described [15].

### Construction of expression vector for VEZT and TCF19

VEZT and TCF19 overexpression vector pEGFP-N1-VEZT and pEGFP-N1-TCF19 were constructed using the overlap PCR or PCR method, respectively and the primers used for two vectors are summarized in Table S1. The PCR products were confirmed by direct DNA sequencing and cloned into the mammalian expression vector pEGFP-N1 as previously described [15]. MKN-45 and NCI-N87 gastric cancer cell lines were used for the overexpression studies. We obtained stably transfected clones by G418 selection (Promega). A stable transfectant of the pEGFP-N1 empty vector was used as a control. For transfection, complexes of Lipofectamine 2000 (Invitrogen Corp, Carlsbad, USA) and one of the plasmids mentioned above was prepared according to the manufacturer’s instructions, and these complexes were directly mixed with cells in 24-well cell culture plates at a density of 4 × 10^4^ cells per well. The level of VEZT or TCF19 expression after transfection was assayed by real-time PCR.

### Invasion, migration and endothelial tube formation assays

Cell invasion, migration and endothelial tube formation assays were performed using MKN-45 and NCI-N87 cells. Cell culture was performed in transwell chambers (Corning, NY, USA). For the invasion assay, the insert membranes were coated with diluted Matrigel (San Jose, CA, USA). Cells (1 × 10^5^) were added to the upper chamber and were cultured for 48 h. For the migration assay, the insert membranes were not coated with Matrigel but were cultured under the same conditions. Finally, the insert membranes were cut and stained with crystal violet (0.04% in water; 100 ml), and the migrated cells were counted under an inverted microscope and were photographed.

In vitro angiogenesis was assessed by using an endothelial tube formation assay kit (San Diego, CA, USA). Briefly, each well of prechilled 96-well culture plates was coated with a thin layer of ECM gel. HUVECs were resuspended in supernatants collected from transfected cells. HUVECs (2 × 10^4^ cells/well) were added to the polymerized ECM gel with 300 mL of the supernatants. After 18-h incubation, the tube formation ability was evaluated by determining the tubular number, the tubular length and the number of tubular intersecting nodes in five random fields using Image Pro Plus software (Media Cybernetics Inc., Bethesda, MD, USA) according to Mirshahi’s method [19].

### Cell growth and Soft agar colony formation assay

Gastric cancer cells (2 × 10^3^ cells) were incubated with 100 µL of culture medium in 96-multiwell plates for one day at 37°C in 5% CO_2_. The cells were transfected with the plasmid for 24, 48, 72, 96, and 120 hours. Cell number was assessed using the cell counting kit-8 (CCK-8) (Dojindo, Japan). Briefly, CCK-8 (10 µL) was added to each well. After 1 h of incubation at 37°, absorbance at 450 nm was measured using the ARVO MX plate reader (PerkinElmer, Massachusetts, USA). The number of cells was determined by the relative absorbance at 450 nm.

Gastric cancer cells were trypsinized to single-cell suspensions of 3 x 10^3^ cells and then were plated in six-well plates in complete culture medium containing 0.3% agar layered on top of 0.6% agar. The plates were incubated at 37°C in the presence of 5% CO_2_ for 16 days. Colonies containing at least 50 cells were scored. The data are presented as the mean ± standard deviation of five randomly scored fields.

### Tumor growth in nude mice

Cells (1 × 10^6^ cells in 100 mL) from cancer cells lines transfected with empty vector or a VEZT expression vector were collected and inoculated subcutaneously into the right flank regions of 4-week-old male BALB/c nude mice (Chinese Academy of Sciences, Shanghai, China). Tumor nodules were measured every 4 days with calipers. Mice were sacrificed after 1 month. Tumor growth curves and growth inhibition rates were calculated. Two mice were used for the experimental and control groups, and three independent experiments were performed as previously described [15,16].

### Global cDNA microarray analysis and target gene verification

Purified total RNA was obtained from the microdissected cells. The whole human genome oligo microarray (Agilent, Santa Clara, CA, USA) was used. After hybridization and washing, the microarray slides were scanned with an Agilent DNA microarray scanner. The resulting text files extracted from Agilent Feature Extraction Software (version 9.5.3) were imported into the Agilent GeneSpring GX software (version 7.3) for further analysis. Differentially expressed genes were identified through fold-change screening. For target gene verification we used real-time PCR and chromatin immunoprecipitation assay. The primers for the target genes are listed in Table S1.

### Flow cytometric analysis of cell cycle

One day before transfection, 1 × 10^6^ cells of MKN-45 cells were seeded into 6-well culture plates without antibiotics. The cells were transfected with empty vector or a TCF19 expression vector. Forty-eight hours after transfection, cells were harvested and fixed in 70% ethanol at -20°C overnight, and then stained with 250 µg/mL propidium iodide (Sigma–Aldrich), 5 µg/mL RNase A (Sigma–Aldrich) and 5 mmol/L EDTA in PBS (pH 7.4) for 30 min. The cell cycle analysis was done by FACScan (Beckman Instruments, Fullerton, CA, USA).

### Statistical analysis

Statistical analysis was performed using SPSS15.0 software (SPSS Inc, USA). Data are expressed as the mean ± standard deviation from at least three separate experiments. The correlation between VEZT expression in the tumors and clinicopathologic variables was calculated with the Kruskal-Wallis rank test and the Mann-Whitney U test. Differences between groups were analyzed using Student’s t-test and Chi-square test. A value of *p* < 0.05 was considered statistically significant.

## Results

### Relationship of VEZT expression levels and clinicopathological factors in patients with gastric cancer

We investigated the relationship between VEZT expression levels in 104 paired gastric cancer tissues and clinicopathological factors in patients with gastric cancer. We found that the expression level of VEZT was associated significantly with lymphatic metastasis, depth of cancer invasion and TNM stage (Table 1, *P* < 0.05). However, VEZT expression levels were not associated with gender, age, tumor size, cell differentiation, gross appearance, site of tumor and distant metastasis.

**Table 1:** Relationship between VEZT expression levels in cancer tissues and clinicopathological factors in patients with gastric cancer.

Factors	No. of patients	Mean expression of VEZT（mean±SD)	*P*-value
Gender			
Male	57	14.87±2.03	1.382
Female	47	15.47±3.15	
Age(year)			
<65	49	18.62±2.15	0.584
≥65	55	13.11±3.45	
Tumor size			
<5cm	51	17.16±3.05	2.682
≥5cm	53	11.26±3.52	
Cell differentiation			
Poor differentiation	68	18.21±1.65	1.827
Moderate differentiation	36	16.6±2.46	
Gross appearance			
Borrmann Ⅰ+Ⅱtype	32	17.25±3.46	2.361
Borrmann Ⅲ+Ⅳtype	72	15.21±6.58	
Site of tumor			
Cardia	21	19.34±2.53	0.692
Body	36	11.24±2.61	
Antrum	47	17.35±2.87	
Lymphatic metastasis			
Positive	74	7.42±7.25	0.027*
Negative	30	18.26±9.32	
Depth of cancer invasion			
T2	25	18.35±7.82	0.041*
T3	63	22.12±4.21	
T4	16	24.78±5.41	
Distal metastasis			
Positive	24	9.34±6.09	1.032
Negative	80	12.35±8.62	
TNM# Stage			
Ⅰ	15	6.35±7.16	0.0385*
Ⅱ	28	16.37±4.56	
Ⅲ	48	17.24±1.41	
Ⅳ	13	7.41±2.36	

We analyzed the relationship between VEZT expression levels in cancer tissues and clinicopathological factors in gastric cancer samples versus adjacent normal tissue (n = 104). We found that the expression level of VEZT was significantly associated with lymphatic metastasis, depth of cancer invasion and TNM stage (Table 1, *P* < 0.05). However, VEZT expression levels were not associated with gender, age, tumor size, cell differentiation, gross appearance, site of tumor and distant metastasis.

#: TNM, tumor-node-metastasis; *p < 0.05.

### Methylation analysis of normal gastric tissues, primary gastric cancer tissues and peripheral blood from gastric carcinoma patients and healthy controls

**Figure 1:**
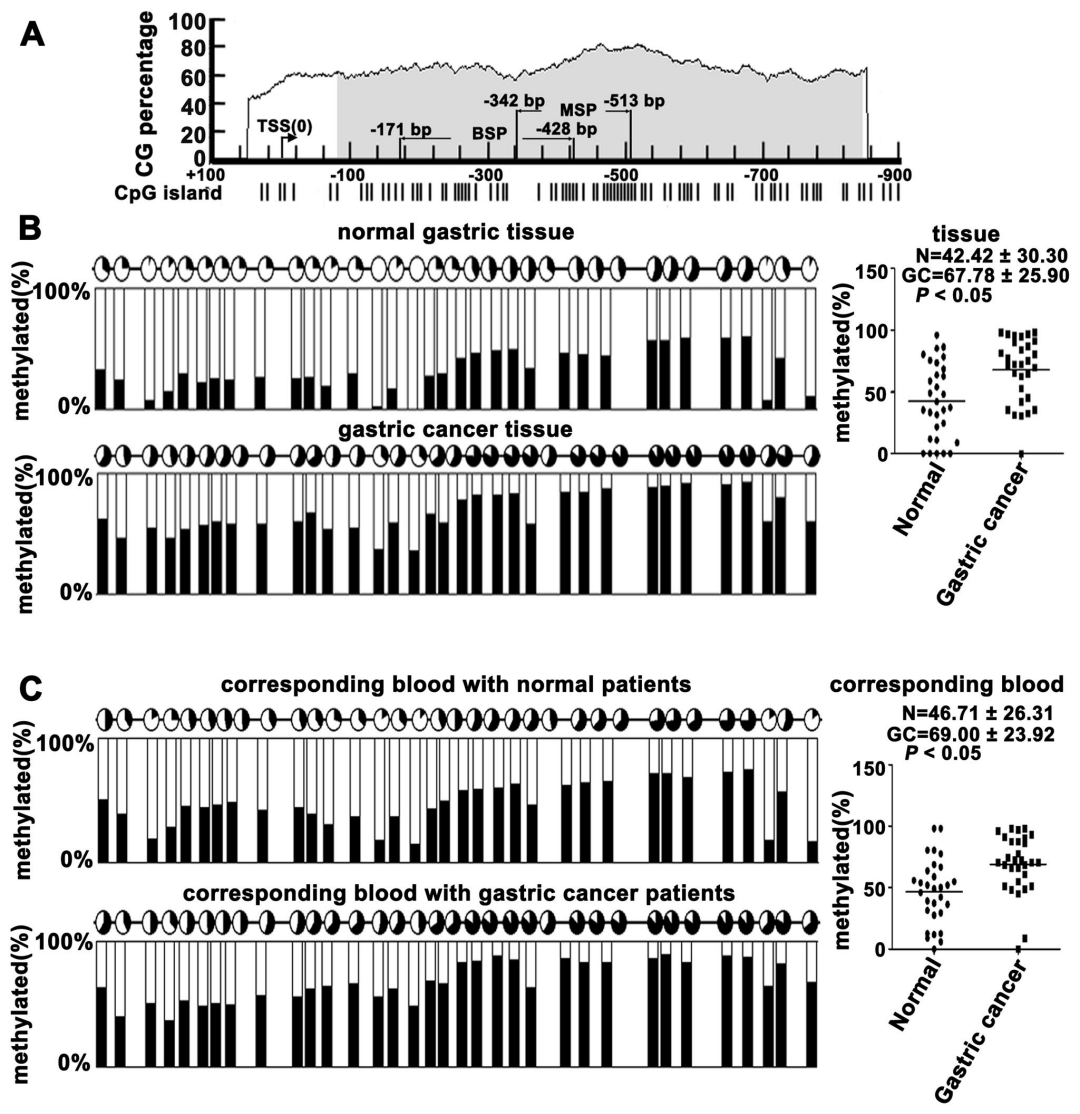
Methylation analysis of normal gastric tissues, primary gastric cancer tissues and peripheral blood from gastric carcinoma patients and healthy controls. (A) Online bioinformatics software was used to analyze the promoter region and the methylation status of the VEZT gene. (B) Methylation status of 34 CpG sites of the VEZT promoter from 30 normal gastric tissues and 30 primary gastric cancer tissues. Primary tumor tissues showed higher methylation levels in the VEZT promoter region when compared with normal gastric tissues. (C) Methylation status of 34 CpG sites of the VEZT promoter from peripheral blood plasma DNA of 30 gastric carcinoma patients and 30 healthy controls. Peripheral blood from gastric carcinoma patients showed higher methylation levels in VEZT promoter region when compared with healthy controls. Each row of circles represents an integrated methylation ratio from three clones, and each circle represents a single CpG site. Open circle represents unmethylated cytosine, whereas filled circles or partially filled circles represent the methylated ratio of CpG sites.

Based on a previous study from our lab, we postulated that the methylation of VEZT promoter was different in gastric carcinoma patients and healthy controls; we used online bioinformatics software to analyze the promoter region and methylation status of the VEZT gene (Figure 1A). We examined the methylation level of the VEZT promoter in 30 tissue samples DNA from patients with primary gastric cancer tissues, corresponding plasma DNA and the control using BSP methods, which covered the regions of -171bp to -428bp (Figure 1A). The mean methylated levels of VEZT in 30 primary gastric cancer tissues and 30 normal gastric tissues were 67.78 ± 25.90% and 42.42 ± 30.30%, respectively (Figure 1B). Primary gastric cancer tissues showed higher methylation levels in the VEZT promoter region when compared with normal gastric tissues (Figure S1A, Figure 1B, P < 0.05). The mean methylated level of plasma DNA in primary gastric cancer patients and healthy control cases were 69.00 ± 23.90% and 46.71 ± 26.31%, respectively (Figure 1C). The methylated level of plasma DNA in primary gastric cancer patients showed higher methylation levels in the VEZT promoter region when compared with healthy control case (Figure S1B, Figure 1C, P < 0.05). Thus, significant methylation was observed in the gastric cancer group compared with healthy controls. The level of methylated VEZT in tissues and plasma could serve as a molecular marker for gastric cancer.

### *H. pylori* infection induces the methylation and silencing of VEZT

**Figure 2:**
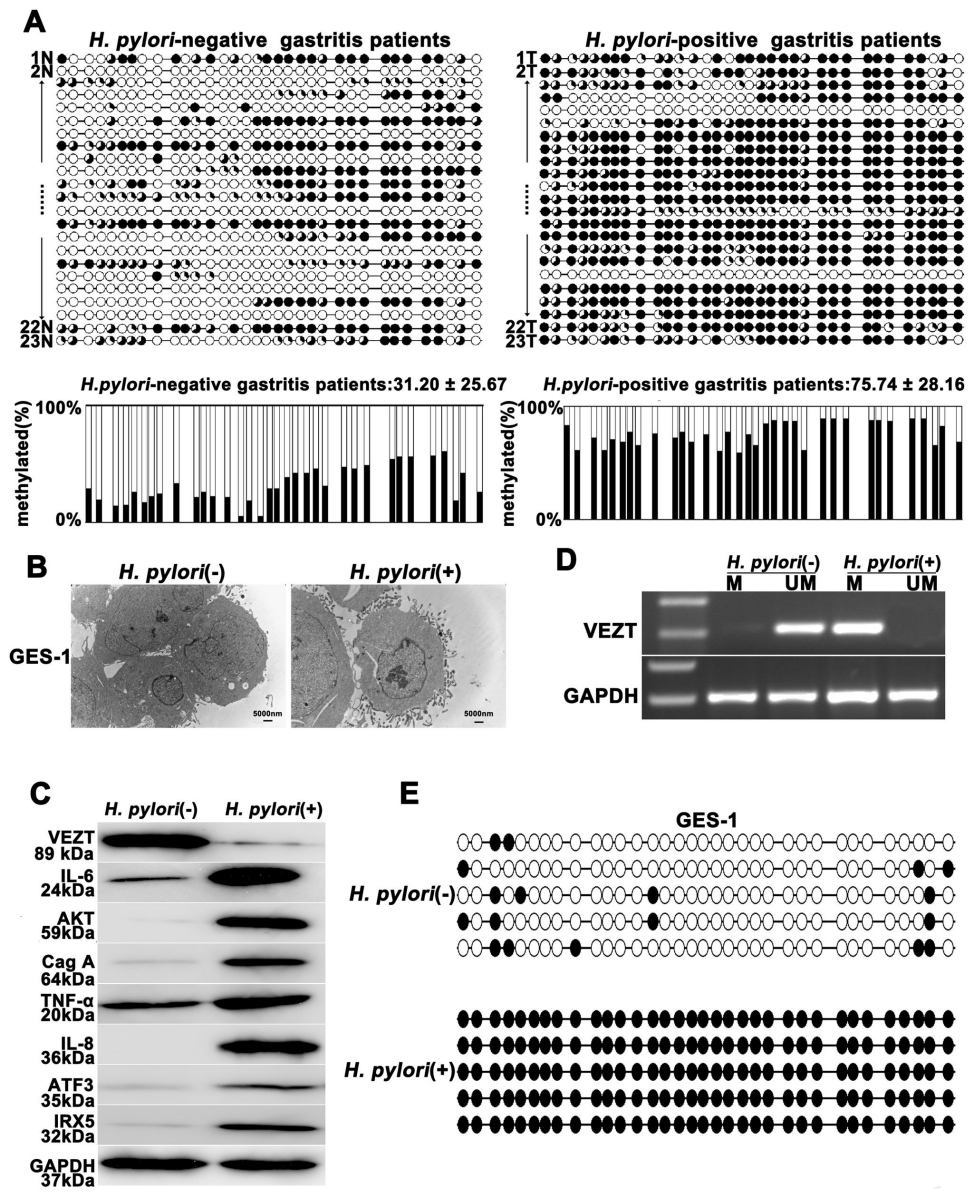
*Helicobacter pylori* infection promotes the methylation and silencing of VEZT. (A) *H. pylori*-positive gastritis patients showed higher methylation levels in the VEZT promoter region when compared with *H. pylori*-negative gastritis patients. Each row of circles represents an integrated methylation ratio from three clones, and each circle represents a single CpG site. Open circle represents unmethylated cytosine, whereas filled circles or partially filled circles represent the methylated ratio of CpG sites. (B) After 24-h infection with *H. pylori*, the attachment of *H. pylori* was observed by transmission electron microscopy on the surface of GES-1 cells in the experimental cells relative to the control cells. (C) VEZT expression level in GES-1 cells was reduced after a 24-h *H. pylori* infection relative to negative control cells; however, *H. pylori* infection induced the IL-6, AKT, TNF-α, IL-8, ATF3 and IRX5 expression by western blot analysis. (D) The methylation of the VEZT promoter was detected by MSP after a 24-h *H. pylori* infection in GES-1 cells (marked as M), whereas methylation of the VEZT promoter in GES-1 cells that were not infected with *H. pylori* was not observed (marked as U). (E) Schematic summary of 34 CpG sites in the promoter region of the VEZT gene from -171 to -428 by BSP analysis. GES-1 cells showed a higher level of methylation after *H. pylori* infection relative to the control samples. The bar represents 5000 nm.

A considerable number of studies have been published demonstrating that *H. pylori* infection is an independent risk factor for methylation [20,21,22]. Therefore, we assume that *H. pylori* causes VEZT methylation and subsequently carcinogenesis. At first, we examined the methylation level of the VEZT promoter in the DNA from 23 tissue samples from patients with *H. pylori*-positive chronic gastritis and the controls using BSP and MSP methods, which covered the regions of -171bp to -428bp and -342 bp to -513 bp, respectively (Figure 1A). The mean methylation levels of the VEZT promoter in *H. pylori*-positive and the control were 75.74 ± 28.16% and 31.20 ± 25.67%, respectively. Thus, a significant difference in methylation was observed in the *H. pylori*-positive chronic gastritis group compared with the control group (*P* < 0.01, Figure 2A). The promoter region of VEZT was generally methylated in patients with chronic gastritis according to MSP analysis (Table S2); however, we found four cases of *H. pylori*-positive chronic gastritis that were relatively unmethylated. We observed 19 cases of *H. pylori*-positive chronic gastritis that displayed hypermethylation (Table S2). To determine whether *H. pylori* infection induces promoter methylation of the VEZT gene *in vitro*, we detected that *H. pylori* infection were associated with the expression of IL-6, AKT, TNF-а, IL-8, ATF3 and IRX5 protein after *H. pylori* infection and incubated for 24 h in GES-1 cells using Western blotting. Our results showed *H. pylori* infection promoted the expression of IL-6, AKT, TNF-а, IL-8, ATF3 and IRX5 protein and *H. pylori* infection was successful in GES-1 cells (Figure 2B, 2C). To further confirm that that *H. pylori* infection is indeed responsible for the methylation or silencing of VEZT, we analyzed the promoter methylation status of VEZT in GES-1 cells by MSP and BSP. As shown in Figure 2D and 2E, the promoter of VEZT was hypermethylation in *H. pylori*-infected GES-1 cells, but unmethylation was observed in cells uninfected with *H. pylori* (Figure 2D, and E). The protein expression level of VEZT was also lower in *H. pylori*-infected cells than in cells uninfected with *H. pylori* (Figure 2C).

### Functional analysis after restoring VEZT expression in vitro

**Figure 3:**
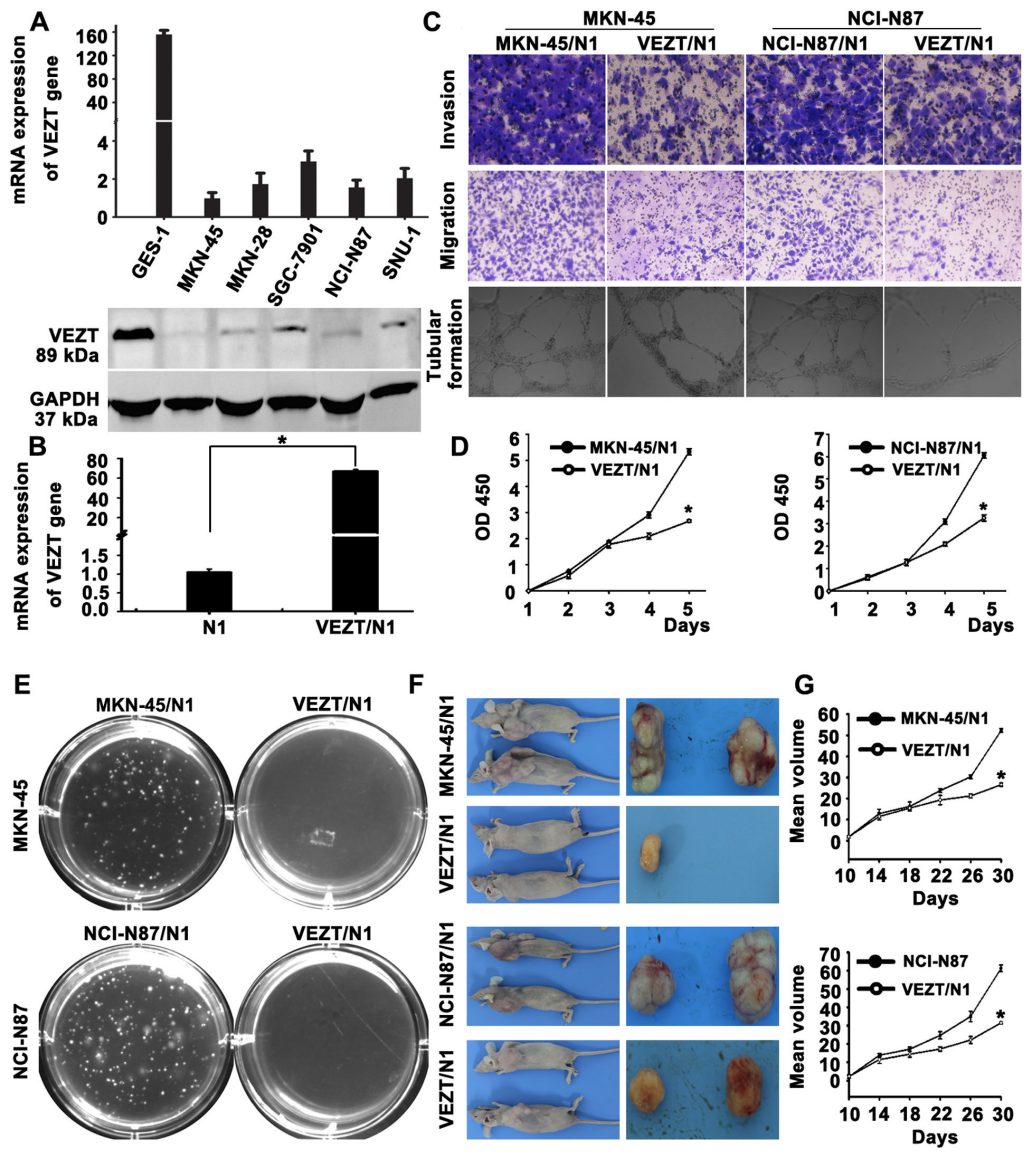
Function of VEZT in gastric cancer cells. (A) The different expression levels of VEZT in five gastric cancer cell lines and one immortalized gastric mucosal cell line. (B) Expression of VEZT was upregulated in MKN-45 and NCI-N87 cells upon VEZT vector transfection relative to N1-controls. (C) Invasive, migratory and tubular formation capacities of VEZT-transfected MKN-45 and NCI-N87 cells were suppressed as determined by the transwell and tubular formation assays. (D) Overexpression of VEZT leads to cell growth arrest as determined by the CCK-8 assay. (E) Colony formation rates were significantly different between VEZT-transfected cells and N1-controls in MKN-45 and NCI-N87 cells. (F) The overexpression of VEZT in MKN-45 and NCI-N87 cells inhibited tumorigenesis in nude mice. Tumor nodules resected from the VEZT-transfected group were smaller than those from N1-controls. (G) Tumor growth curves for the VEZT/N1 group and N1-controls show rapid tumor growth in the MKN-45 or NCI-N87/N1control groups. *P < 0.05. Each bar represents the mean value ± standard deviation from three independent experiments.

The frequent silencing or downregulation of VEZT in gastric cancer cell lines suggests that it is likely a tumor-suppressor gene in our previous study. In order to test this point, we screened VEZT expression in several gastric cancer cell lines by real-time PCR and western blot. Different VEZT expression levels were found in the six cell lines analyzed (Figure 3A). We constructed a pEGFP-N1-VEZT expression vector and transfected pEGFP-N1 vector into the gastric cancer cell line MKN-45 and NCI-N87, which showed no or low level of VEZT expression. After transfection, we examined the expression of VEZT in these cell lines by real-time PCR, which showed that the transcript expression level of VEZT was up-regulated 41.99-fold (Figure 3B, P < 0.01) in these cell lines compared to with the control-transfected cells. We also examined the capacity of gastric cancer cells overexpressing VEZT to invade and migrate by the transwell invasion and migration assays (Figure 3C). The invasive ability of MKN-45 cells in the VEZT/N1 group was significantly reduced when compared with the control-transfected cells (78.32 ± 5.27 vs. 174.13 ± 2.45, *P* < 0.001). The number of MKN-45 cells in the VEZT-transfected sample that migrated through the transwell insert was also significantly reduced when compared with the control-transfected cells (48.28±2.16 vs. 142.13±7.42, *P* < 0.001).

HUVECs were suspended in supernatants collected from the control and VEZT/N1. After 18-h incubation, the supernatant harvested from VEZT/N1 cells showed a strong inhibitory effect on the formation of tubular structures by HUVECs with respect to the number, length and intersecting nodes when compared with the control group (Figure 3C). As shown in Figure 3D, the cell growth of MKN-45 cells was significantly suppressed upon the restoration of VEZT expression relative to the control group (*P* < 0.05). To examine the reproducibility of our findings, we assessed the ability of NCI-N87 gastric cancer cells to invade, migrate and form tubular structures upon VEZT overexpression. These results were similar to those described in MKN-45 cancer cells, which suggest that VEZT has an important inhibitory role in the invasion, migration and tubular formation of cancer cells.

### Effect of VEZT overexpression on the tumorigenicity of cancer cells in nude mice

**Figure 4:**
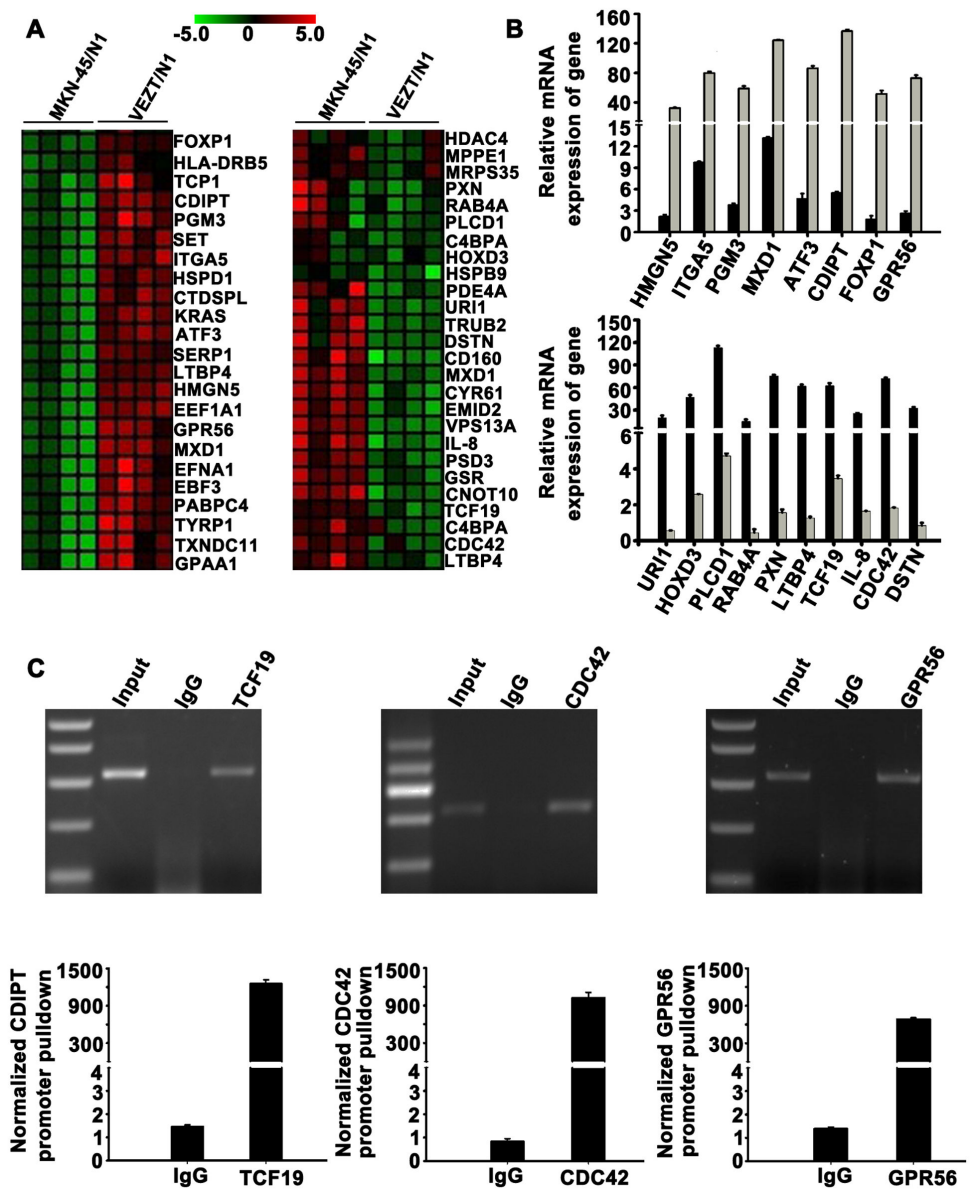
Target gene identification by global microarray analysis. (A) Clustering map of differentially expressed genes overlapped with cancer-associated genes set in the Molecular Signatures Database. Row represents gene, column represents experimental cells. Upregulated genes are shown in red (left) and downregulated genes in green (right). (B) Upregulation of HMGN5, ITGA5, PGM3, MXD1, ATF3, CDIPT, FOXP1 and GPR56 were confirmed (up). Downregulation of URI1, HOXD3, PLCD1, RAB4A, PXN, LTBP4, TCF19, IL-8, CDC42 and DSTN were confirmed (down). (c) Chromatin immunoprecipitation analysis was done with VEZT antibody using lysates from MKN-45 cells. Promoter pulldowns were assessed through quantitative PCR, which revealed amplified GPR56, TCF19 and CDC42 compared with the negative IgG control.

Expression of VEZT suppressed the growth of both MKN-45 and NCI-N87 by CCK-8 assay when compared to the control group (Figure 3D, P < 0.05). This finding was further confirmed by colony formation assay, by growing gastric cancer cells on soft agar after transfection with a VEZT-overexpressing vector. Colony formation rates of MNK-45 cells were 13.56 ± 0.53% in the VEZT/N1 group and 1.2 ± 0.31% in the control group (*P* < 0.001, Figure 4E). The colony formation rate of NCI-N87 cells showed a similar trend. Next, we examined the effect of VEZT overexpression on tumor growth by inoculating MKN-45 or NCI-N87/N1 and VEZT/N1 cells subcutaneously into the right flank regions of nude mice. Tumorigenicity was significantly reduced in VEZT-transfected cells. Rapid tumor growth was observed in the control groups after 1 month (Figure 4F). The tumor suppressive role of VEZT expression was apparent in both cancer cell lines tested (Figure 4G, P < 0.01), suggesting that VEZT suppresses the tumorigenicity of cancer cells. These results indicated that the expression of VEZT inhibited the tumorigenicity of gastric cancer both *in vitro* and *in vivo*.

### Identification of target genes after VEZT gene transfection

To elucidate the molecular mechanism underlying the inhibitory effect of VEZT on cells invasion, growth, migration and the tumorigenicity of gastric cancer. We analyzed the genome-wide transcriptome profile of MKN-45/N1 and VEZT /N1 cells by Agilent oligo microarray. According to fold-change (>3.0), screening between MKN-45/N1 and VEZT/N1 cells, we found 193 upregulated genes and 135 downregulated genes (Table S3). We searched genes that overlapped with cancer-associated and molecular function-related gene sets in MSigDB (C4 and C5 gene sets; http://www.broad.mit.edu/gsea/msigdb/index.jsp). We selected 49 cancer-associated genes that include 23 upregulated genes and 26 downregulated genes for cluster mapping on the MeV microarray analysis platform (www.tm4.org/mev.html, Figure 4A). Real-time PCR was performed for verification of these genes (Table S4) and confirmed our microarray findings for 8 upregulated genes and 10 downregulated genes (Figure 4B). The gene names and functional annotations are listed in Table S5. To determine whether these are direct target genes of VEZT, we conducted chromatin immunoprecipitation assays using VEZT antibody and then analyzed the pulled-down DNA. We identified three downregulated genes, TCF19 (NM_001077511), CDC42 (NM_001039802) and GPR56 (NM_001145770) as direct VEZT targets (Figure 4C).

**Figure 5:**
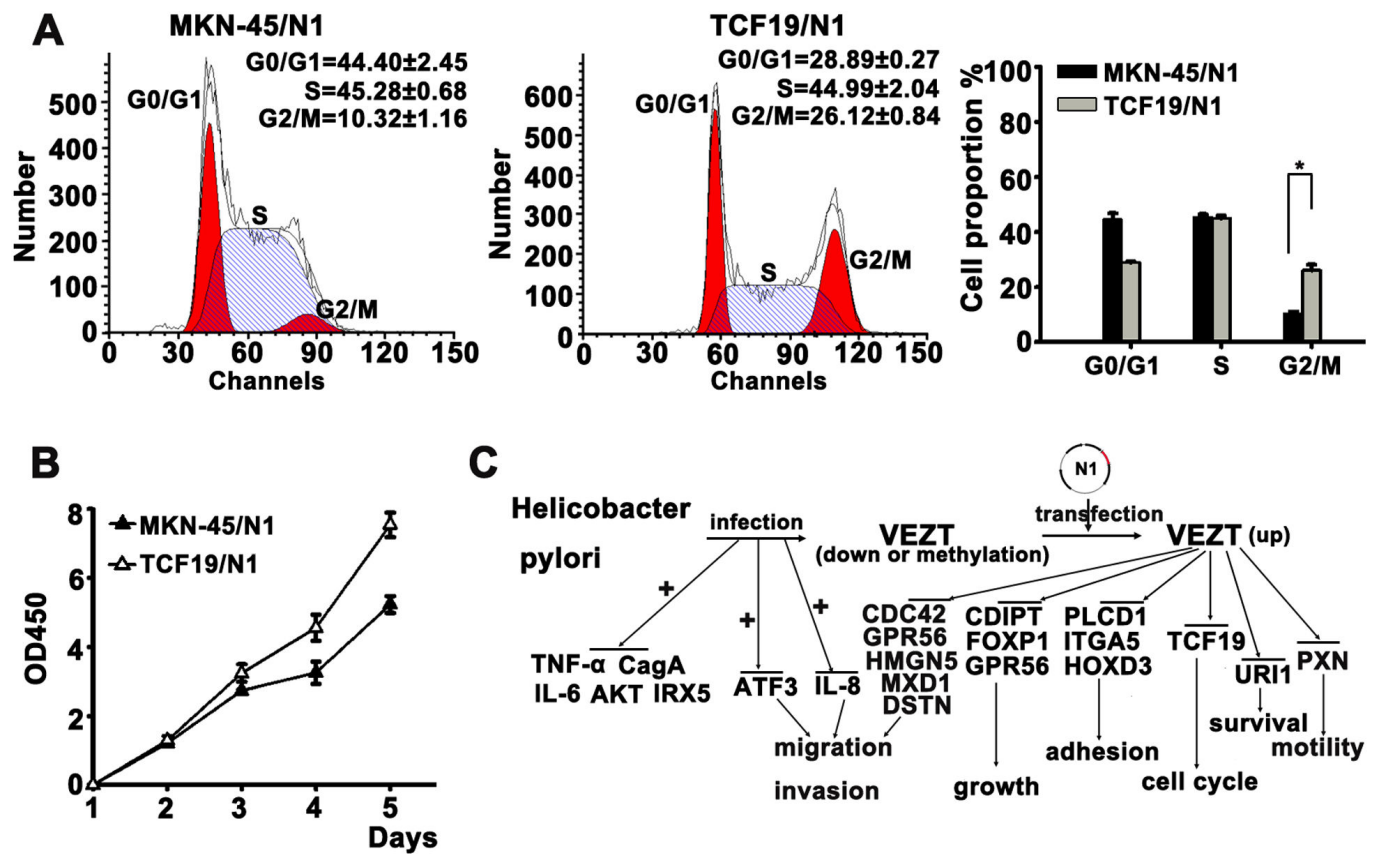
Molecular mechanism of the inhibitory activity of VEZT on gastric cancer growth. (A) TCF19-transfected MKN-45 showed increased percentage of G2/M phase cells and decreased percentage of S and G0/ G1 phase cells. (B) Over-expression of TCF19 promoted cell growth by CCK-8 assay. (C) Schematic summary of the VEZT tumor suppressor gene on carcinogenesis of gastric cancer. VEZT is inactivated via hypermethylation, which may be induced by *H. pylori* infection. Restoring VEZT expression inhibits cell proliferation, migration, invasion and tumorigenesis both in vitro and in vivo, which could be explained by the downregulation of specific target genes identified by global microarray analysis. *P < 0.05. Each bar represents the mean value ± standard deviation from three independent experiments.

The correlations between VEZT and its downstream targets, as well as their association with the inhibition of gastric cancer cells invasion, growth, migration and the tumorigenicity of gastric cancer *in vitro* and *in vivo*, were shown in Figure 5C. These results unveiled the molecular mechanism by which VEZT inhibited the growth and invasiveness of cancer cells.

### Functional validation of a novel VEZT downstream target TCF19

We further selected TCF19, a novel downstream target of VEZT, for further functional validation. We analyzed the effect of TCF19 on cell cycle distribution by flow cytometry. Ectopic expression of TCF19 led to a significant increase in the number of the G2/M-phase cells of MKN-45 as compared with MKN-45/N1-transfected cells, TCF19-transfected MKN-45 showed higher percentage of G2/M phase cells and lower percentage of G0/ G1 phase or S phase cells (*P* < 0.05, Figure 5A), confirming that over-expression of TCF19 promoted G2/M transition. In addition, ectopic expression of TCF19 in MKN-45 cells significantly enhanced MKN-45 cell growth as early as 120 h post-transfection as compared with empty vector-transfected MKN-45 cells (*P* < 0.05, Figure 5B). These results indicated that VEZT inhibited gastric cancer growth and invasion, at least in part, by downregulation of TCF19.

## Discussion

Gastric cancer is the second most common cancer in the world. It is now apparent that multiple genetic alterations, including *Helicobacter pylori* infection, oncogene activation and tumor suppressor gene inactivation, are necessary steps in gastric cancer development [23,24,25,26,27]. In a previous study, we revealed VEZT was downregulated in gastric tumor samples by RT-PCR, the down-expression levels of VEZT was further revealed to be associated with methylation [13]. Prior to this study, very little was known about VEZT expression in gastric carcinoma and its correlation with the clinicopathologic features of these patients. To address these questions, VEZT expression levels and the clinicopathologic characteristics of 104 patients with gastric cancer were examined. While there was no relationship between VEZT expression and gender, age, tumor size, cell differentiation, gross appearance, site of tumor and distant metastasis, there was a significant association between VEZT expression and lymphatic metastasis, depth of cancer invasion and TNM stage. These results suggested that the expression levels of VEZT may be involved in tumor progression during gastric cancer development. Promoter methylation was demonstrated to mediate the transcriptional silence of VEZT gene in gastric cancer cell lines. Epigenetic events, especially promoter methylation, are now widely accepted as the cause of gene silencing [28,29,30,31]. We further analyzed the methylation of VEZT in tissues and corresponding blood of gastric cancer patients. Using bisulfate sequencing polymerase chain reaction methods, the results showed that VEZT was hypermethylated in gastric cancer patients when compared with healthy controls.

*H. pylori* infection is a major causative factor for gastric cancer, chronic gastritis, peptic ulcers and atrophic gastritis [2,32,33]. The risk of patients with *H. pylori* infection developing gastric cancer is of two- to six-fold according to most retrospective case control and prospective epidemiologic studies [32]. Several factors have been reported which affect gastric carcinogenesis by *H. pylori* infection [22,34,35,36,37]. Current reports have demonstrated that high levels of aberrant methylation of several CpG islands were isolated in *H. pylori* infected gastric mucosae and its possible association with gastric cancer risk [38]. Many studies have suggested that *H. pylori* infection induces the methylation of tumor suppressor genes such as E-cadherin, runx3, IRX1, TFF2 and p16 [22,38,39,40,41,42,43]. However, whether or not *H. pylori* infection directly induces VEZT methylation in the gastric mucosa remains to be elucidated. In this study, we analyzed the effect of *H. pylori* infection on the methylation status of the VEZT promoter. We found a strong association between the methylation of the promoter of the VEZT gene and *H. pylori* infection. The promoter methylation status of VEZT in *H. pylori*-positive gastritic samples was approximately 2.4-fold higher than the control samples. Some studies have demonstrated that upon infection, *H. pylori* activates the expression of a number of genes such as CagA, AKT, TNF-α, ATF3, IRX5, IL-6 and IL-8. In *H. pylori*-infected GES-1 cells, our results show that persistent infection with *H. pylori* can promote the expression of CagA, AKT, TNF-α, ATF3, IRX5, IL-6 and IL-8 protein and induce VEZT promoter methylation as determined by BSP and MSP and cause the significant downregulation of VEZT gene expression. Indeed, an increasing amount of evidence suggests that the promoter hypermethylation of tumor suppressor genes in the gastric mucosa is closely correlated with an increased risk of gastric carcinogenesis. VEZT is one of the hypermethylated genes associated with *H. pylori* infection in the gastric mucosa. Our study is the first to demonstrate a link between *H. pylori* infection and VEZT promoter methylation.

Only limited data existed regarding a functional association between the VEZT gene and human cancer. In cell biology experiments, we constructed a VEZT-expressing vector and restored VEZT expression in MKN-45 and NCI-N87 gastric cancer cells for gain-of-function study. These experiments revealed that restoring VEZT can reduce the invasive and migratory capacity of gastric carcinomas *in vitro*. Proliferation and tumorigenicity assays revealed that VEZT overexpression inhibits the growth and tumorigenicity of MKN-45 and NCI-N87 cells both *in vitro and in vivo*. These results indicate for the first time that VEZT functions as a tumor suppressor in gastric cancer.

To clarify the possible mechanism of VEZT’s tumor-inhibiting function, we screened the target genes regulated by VEZT. cDNA microarrays provide a powerful tool for exploring complex gene expression profiles. Microarray analysis of experimental samples, such as gene-transfected cells, has led to identification of valuable molecular markers involved in tumor proliferation, invasion, migration, prognosis and therapeutic response [44,45,46]. Thus, we used a global cDNA microarray to identify downstream target genes of VEZT. We compared changes in gene expression profiles in gastric cancer cells with or without VEZT gene transfection and identified a set of VEZT target genes. Using real-time PCR analysis, we confirmed 8 upregulated genes and 10 downregulated genes. Subsequently, we identified three direct VEZT target genes by chromatin immunoprecipitation assay: GPR56, CDC42 and TCF19. GPR56 encodes a member of the G protein-coupled receptor family and overexpression of this gene can suppress tumor growth and metastasis. It was reported that GPR56 interacts with extracellular matrix and regulates cancer progression [47,48,49]. CDC42 encoded a small GTPase of the Rho-subfamily, which regulates signaling pathways that control diverse cellular functions including cell morphology, migration, endocytosis and cell cycle progression [50,51,52]. TCF19 gene can be considered as coding for a new trans-activating factor that could play an important role in the transcription of genes required for the later stages of cell cycle progression [53]. These findings raised the possibility that VEZT overexpression downregulated the expression of CDC42 and TCF19, upregulated the expression of GPR56, consequently inhibiting invasion, migration and cell proliferation in gastric cancer. Of course, other indirect target genes, such as the forkhead box P1(FOXP1) gene, belongs to subfamily P of the forkhead box (FOX) transcription factor family, have been reported to act as a candidate tumor suppressor gene localized to the chromosome 3p14, 1 region [54,55]. We further selected TCF19, a novel downstream target of VEZT, for further functional validation. Our results found that ectopic expression of TCF19 promoted cell cycle progression. In addition, ectopic expression of TCF19 enhanced gastric cancer cell growth. These results indicated that VEZT inhibited gastric cancer growth and invasion, at least in part, by downregulation of TCF19.

In conclusion, we have outlined the functions of VEZT, tumor suppressor gene during gastric carcinogenesis in Figure 5C. VEZT expression is markedly downregulated and regulated epigenetically by DNA methylation in gastric cancer cells. *H. pylori* infection induces the methylation of VEZT. Restoring VEZT expression inhibits cell proliferation, migration, invasion and tumorigenesis both *in vitro and in vivo*. The mechanism of the tumor suppressor effect of VEZT overexpression could be explained by a set of target genes identified by global microarray analysis and confirmed by real-time PCR and chromatin immunoprecipitation assay. VEZT suppressed the proliferation, migration, invasion and tumorigenesis of gastric cancer cells, which involved the cell migration and invasion genes (CDC42,GPR56,HMGN5, MXD1 and DSTN) and growth genes (CDIPT,FOXP1, GPR56), as well as inducing cellular adhesion genes (PLCD1, ITGA5 and HOXD3) and a cell cycle progression gene (TCF19). VEZT possesses important functions for the suppression of gastric cancer. Hypermethylation of VEZT detected in the peripheral blood of gastric cancer patients suggests a biomarker potential for gastric cancer. Restoring VEZT activity may be a potential strategy for gastric cancer treatment.

## Supporting Information

Figure S1 Methylation analysis of primary tumor tissues and peripheral blood from gastric carcinoma patients and healthy controls. (A) Methylation status of 34 CpG sites of the VEZT promoter from primary tumor tissues from 30 gastric carcinoma patients and age-matched healthy controls. (B) Methylation status of 34 CpG sites of the VEZT promoter from plasma DNA from 30 gastric carcinoma patients and age-matched healthy controls. Each row of circles represents an integrated methylation ratio from three clones, and each circle represents a single CpG site. Open circle represents unmethylated cytosine; filled circle or partially filled circle represents methylated ratio.
(TIF)

Table S1 We listed all primers in our manuscript.
(DOC)

Table S2 Characteristics and methylation status of 23 *H. pylori*-positive chronic gastritis and the controls patients. We examined the methylation level of the VEZT promoter in the DNA from 23 tissue samples from patients with *H. pylori*-positive chronic gastritis and the controls using MSP methods. Thus, a significant difference in methylation was observed in the *H. pylori*-positive chronic gastritis group compared with the control group.
(DOC)

Table S3 Upregulated genes and downregulated genes were identified by global microarray analysis.
(DOC)

Table S4 23 Upregulated and 26 downregulated target genes were identified by global microarray analysis.
(DOC)

Table S5 Verified genes and their functions by real-time PCR. We examined 8 Upregulated and 10 downregulated target genes by real-time PCR and listed their funtions.
(DOC)
