# Supplementary figures and images for: VEZT, a Novel Putative Tumor Suppressor, Suppresses the Growth and Tumorigenicity of Gastric Cancer
Source: PLoS One. 2013 Sep 17;8(9):e74409. doi: 10.1371/journal.pone.0074409 (PMC3775783; doi:10.1371/journal.pone.0074409)

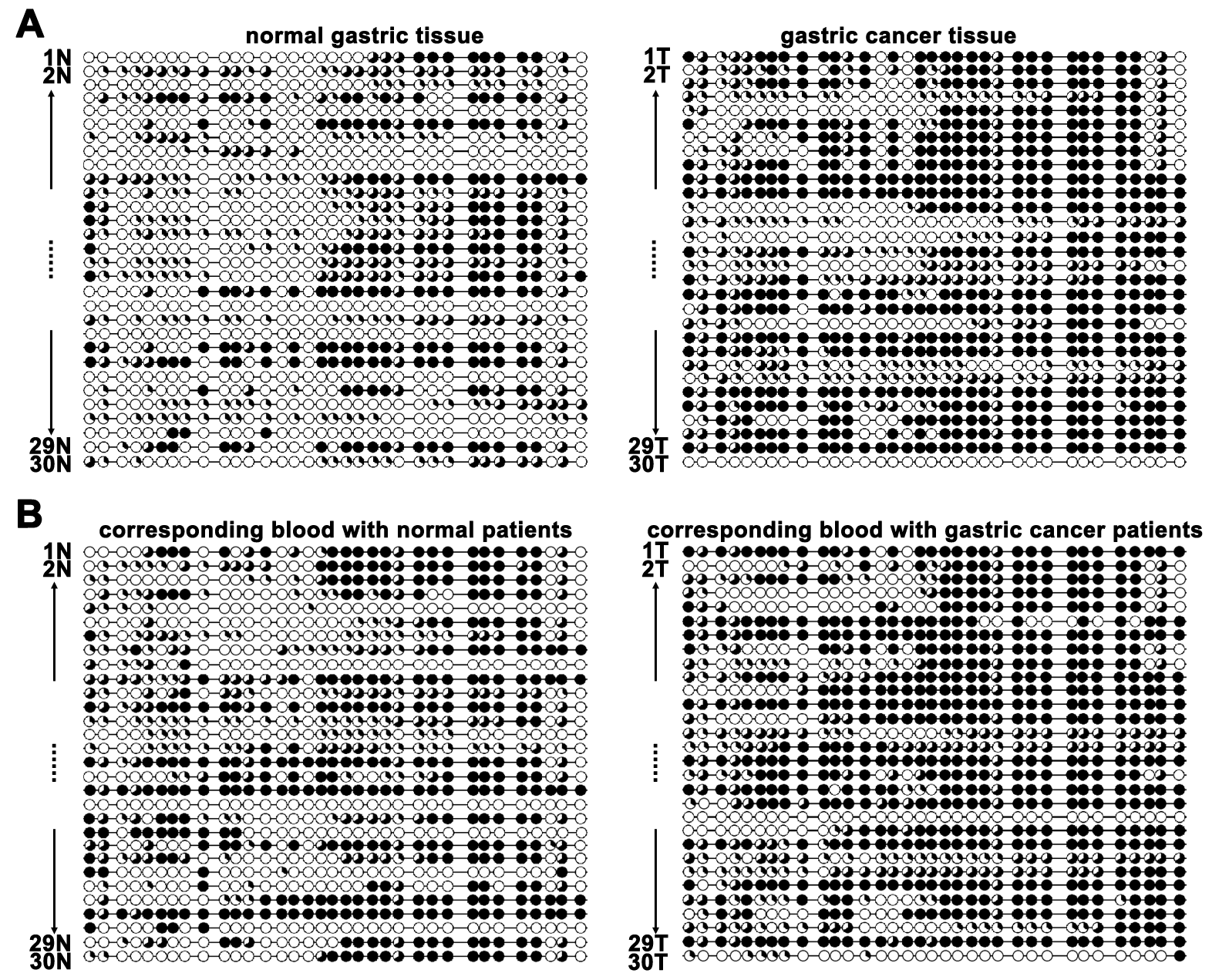

Supplement: Figure S1 — Methylation analysis of primary tumor tissues and peripheral blood from gastric carcinoma patients and healthy controls. (A) Methylation status of 34 CpG sites of the VEZT promoter from primary tumor tissues from 30 gastric carcinoma patients and age-matched healthy controls. (B) Methylation status of 34 CpG sites of the VEZT promoter from plasma DNA from 30 gastric carcinoma patients and age-matched healthy controls. Each row of circles represents an integrated methylation ratio from three clones, and each circle represents a single CpG site. Open circle represents unmethylated cytosine; filled circle or partially filled circle represents methylated ratio. (TIF) [file pone.0074409.s001.tif]
