# Supplementary material for: VEZT, a Novel Putative Tumor Suppressor, Suppresses the Growth and Tumorigenicity of Gastric Cancer
Source: PLoS One. 2013 Sep 17;8(9):e74409. doi: 10.1371/journal.pone.0074409 (PMC3775783; doi:10.1371/journal.pone.0074409)
Supplement: Table S1 — We listed all primers in our manuscript. (DOC) [file pone.0074409.s002.doc]

| primer | | | |
| --- | --- | --- | --- |
| Name | Sequence | Length(bp) | Annealing (^0^C） |
| RT-F | ACTGCCCCTCCAACTCCCAGG | 106 | 58 |
| RT-R | CTGCAGCAAGGCCAGCAGTGA |
| GAPDH-F | GGACCTGACCTGCCGTCTAG | 100 | 58 |
| GAPDH-R | GTAGCCCAGGATGCCCTTGA |
| BSP-F | GGGTAGGAGAGAAAAGTTTT | 257 | 60 |
| BSP-R | AAACACTACTAATTTTTAAAACCC |
| M-F | GAAGTTATGATTTTTCGGTGTAGTTC | 171 | 60 |
| M-R | GACCGCGTCCTTTATATAACG |
| UM-F | GTTATGATTTTTTGGTGTAGTTTGT | 170 | 59 |
| UM-R | TCAACCACATCCTTTATATAACACT |
| UTR-F | CAC ACTAGT TAAGATGTGGATTTA  CAGGAAG | 598 | 58 |
| UTR-R | CTC AAGCTT CCACTTTGACATCAT  CACTTTC |
| hVEZT-SacI-NF | TATGAGCTCATGACACCGGAGTTT  GACGA | 1263 | 61 |
| VEZT-SOE1-reverse | CTGTTGAGTTTTGGATAAACACTGCGG |
| hVEZT-AgeI-NR | GACACCGGTTTACTTTTCTTCTATCTCA  TTTTTATTTTC | 1122 | 62 |
| VEZT-SOE2-forward | CCGCAGTGTTTATCCAAAACTCAACAG |
| TCF19-N1-F | ACTAGATCTATGCTGCCCTGCTTCCA  ACTG | 1038 | 63 |
| TCF19-N1-R | TCTGAATTCTTAGGTCTGAATGCCAG  CCCG |
| HMGN5-RT-F | TAGCACCGCGAGATCTGTTG | 221 | 59 |
| HMGN5-RT-R | ACGACCTGTACTCTCCTAGCTT |
| ITGA5-RT-F | GGCCAAGACTTTCTTGCAGC | 128 | 62 |
| ITGA5-RT-R | GCCACCTGACGCTCTTTTTG |
| PGM3-RT-F | AGGGACCCAGGATAAGGTTGA | 251 | 61 |
| PGM3-RT-R | GCCTTCGTTCGAAATCCAGC |
| MXD1-RT-F | CCGACTCCGACAGGGAAATC | 234 | 62 |
| MXD1-RT-R | AACCTTCAAGGAGACAGCCG |
| ATF3-RT-F | TTTTCAGCACCTTGCCCCAA | 1122 | 60 |
| ATF3-RT-R | GGGGCTACCTCGGCTTTTG |
| CDIPT-RT-F | TGCCCAACCTCATCGGTTAT | 217 | 58 |
| CDIPT-RT-R | TTGACCAACAGGCACATGGT |
| FOXP1-RT-F | ATCAGCCCTCTAGGAGTCCC | 72 | 62 |
| FOXP1-RT-R | GCAGGACTTCCAACTCCCAA |
| GPR56-RT-F | TCTGCTCCAGGAGGAAACCT | 172 | 62 |
| GPR56-RT-R | CAGGTGAGCAGGGAGAAGTG |
| URI1-RT-F | TCTGGCATATGCATTGGTGGT | 109 | 58 |
| URI1-RT-R | ATCAGGCAAGGTGCTGAGTC |
| HOXD3-RT-F | TAATTGTGGTCACCTGGAGCCT | 117 | 62 |
| HOXD3-RT-R | CCTGCTGACCCTGCTCAAAT |
| PLCD1-RT-F | CAGAGCAGAGGGTGTTGTGA | 199 | 61 |
| PLCD1-RT-R | ATCATCCTGTAGGCCCAGCC |
| RAB4A-RT-F | ATGTCCGAAACCTACGGTCC | 112 | 62 |
| RAB4A-RT-R | TCACAATGTTCTGGCTCGCT |
| PXN-RT-F | CATGGACGACCTCGACGC | 76 | 60 |
| PXN-RT-R | CAAGAACACAGGCCGTTTGG |
| LTBP4-RT-F | TGCATCCGAGCCTTCTGC | 273 | 62 |
| LTBP4-RT-R | GATCAAGGGACACAGGACCG |
| TCF19-RT-F | CGCCGCGCGGATTGG | 108 | 60 |
| TCF19-RT-R | CAGGAAGGTCTCCGTGCAAT |
| IL-8-RT-F | GCTCTGTGTGAAGGTGCAGTT | 203 | 58 |
| IL-8-RT-R | ACCCAGTTTTCCTTGGGGTC |
| CDC42-RT-F | TGATACTGCAGGGCAAGAGG | 291 | 59 |
| CDC42-RT-R | TTGACAGCCTTCAGGTCACG |
| DSTN-RT-F | ACCCACTAGAGGCAAAGACG | 133 | 61 |
| DSTN-RT-R | CACTCCTGAGGCCCTAAAGC |
| GPR56-P-F | CCTTGGGCAACGTGGGAACAC | 291 | 62 |
| GPR56-P-R | GGCCAGCCTGTCCCTTCTCTGG |
| TCF19-P-F | CCTCCCAGGACCCCCGCTCAGCC | 275 | 60 |
| TCF19-P-R | GGAGCCGCCTGGGCGCGGAGATGC |
| CDC42-P-F | AGGTGGATCACTTACTTGAGG | 150 | 62 |
| CDC42-P-R | GCGATCCTCCTGCCTCTGC |
