# Supplementary material for: VEZT, a Novel Putative Tumor Suppressor, Suppresses the Growth and Tumorigenicity of Gastric Cancer
Source: PLoS One. 2013 Sep 17;8(9):e74409. doi: 10.1371/journal.pone.0074409 (PMC3775783; doi:10.1371/journal.pone.0074409)
Supplement: Table S2 — Characteristics and methylation status of 23 H. pylori-positive chronic gastritis and the controls patients. We examined the methylation level of the VEZT promoter in the DNA from 23 tissue samples from patients with H. pylori-positive chronic gastritis and the controls using MSP methods. Thus, a significant difference in methylation was observed in the H. pylori-positive chronic gastritis group compared with the control group. (DOC) [file pone.0074409.s003.doc]

| Table 2 Patients,characteristics and methylation status | | | | |
| --- | --- | --- | --- | --- |
|  |  |  |  |  |
| Patients | Sex | Age | H.pylori | methylation |
| 1 | F | 56 | Positive | M |
|  | F | 63 | Negative | UM |
| 2 | M | 47 | Positive | M |
|  | F | 34 | Negative | UM |
| 3 | F | 21 | Positive | M |
|  | M | 47 | Negative | UM |
| 4 | M | 87 | Positive | M |
|  | M | 48 | Negative | UM |
| 5 | F | 30 | Positive | UM |
|  | M | 74 | Negative | M |
| 6 | M | 46 | Positive | UM |
|  | F | 27 | Negative | M |
| 7 | F | 39 | Positive | M |
|  | M | 54 | Negative | UM |
| 8 | F | 46 | Positive | M |
|  | M | 71 | Negative | UM |
| 9 | M | 53 | Positive | M |
|  | M | 28 | Negative | UM |
| 10 | M | 47 | Positive | M |
|  | M | 32 | Negative | UM |
| 11 | M | 24 | Positive | M |
|  | F | 70 | Negative | UM |
| 12 | M | 65 | Positive | M |
|  | M | 54 | Negative | UM |
| 13 | M | 34 | Positive | M |
|  | F | 52 | Negative | UM |
| 14 | M | 36 | Positive | UM |
|  | M | 35 | Negative | M |
| 15 | M | 67 | Positive | M |
|  | M | 18 | Negative | UM |
| 16 | F | 20 | Positive | M |
|  | M | 32 | Negative | UM |
| 17 | M | 35 | Positive | M |
|  | F | 41 | Negative | UM |
| 18 | M | 42 | Positive | M |
|  | M | 57 | Negative | UM |
| 19 | M | 53 | Positive | UM |
|  | M | 24 | Negative | UM |
| 20 | M | 45 | Positive | M |
|  | M | 37 | Negative | UM |
| 21 | M | 38 | Positive | M |
|  | M | 65 | Negative | UM |
| 22 | F | 83 | Positive | M |
|  | F | 79 | Negative | UM |
| 23 | M | 37 | Positive | M |
|  | F | 32 | Negative | UM |
