# Supplementary material for: VEZT, a Novel Putative Tumor Suppressor, Suppresses the Growth and Tumorigenicity of Gastric Cancer
Source: PLoS One. 2013 Sep 17;8(9):e74409. doi: 10.1371/journal.pone.0074409 (PMC3775783; doi:10.1371/journal.pone.0074409)
Supplement: Table S3 — Upregulated genes and downregulated genes were identified by global microarray analysis. (DOC) [file pone.0074409.s004.doc]

| **Fold Change** | **Genbank** | **GeneSymbol** | **Regulation** |
| --- | --- | --- | --- |
| 14.87 | NM_001199917 | PGM3 | up |
| 10.52 | NM_014445 | SERP1 | up |
| 9.441 | NM_017434 | DUOX1 | up |
| 7.817 | NM_001402 | EEF1A1 | up |
| 7.484 | NM_003011 | SET | up |
| 7.181 | NM_031313 | ALPPL2 | up |
| 6.769 | NM_002125 | HLA-DRB5 | up |
| 6.725 | NM_030752 | TCP1 | up |
| 6.68 | NM_003011 | SET | up |
| 6.527 | NM_003819 | PABPC4 | up |
| 6.412 | NM_002156 | HSPD1 | up |
| 6.358 | NM_030763 | HMGN5 | up |
| 6.275 | NM_033360 | KRAS | up |
| 6.211 | NM_015914 | TXNDC11 | up |
| 5.924 | NM_001005463 | EBF3 | up |
| 5.698 | NM_021998 | ZNF6 | up |
| 5.641 | NM_003819 | PABPC4 | up |
| 5.59 | NM_000550 | TYRP1 | up |
| 5.354 | NM_003801 | GPAA1 | up |
| 5.326 | NM_020342 | SLC39A10 | up |
| 5.303 | NM_003564 | TAGLN2 | up |
| 5.277 | NM_145351 | SCARF1 | up |
| 5.237 | NM_030940 | HBLD2 | up |
| 5.218 | NM_003944 | SELENBP1 | up |
| 5.144 | NM_017758 | FLJ20308 | up |
| 5.1 | NM_001986 | ETV4 | up |
| 5.048 | NM_001235 | SERPINH1 | up |
| 5.041 | NM_001005328 | OR2A7 | up |
| 5.033 | NM_003801 | GPAA1 | up |
| 4.965 | NM_001844 | COL2A1 | up |
| 4.845 | NM_031989 | PCBP2 | up |
| 4.768 | NM_020452 | ATP8B2 | up |
| 4.761 | NM_000847 | GSTA3 | up |
| 4.754 | NM_032704 | TUBA6 | up |
| 4.644 | NM_175883 | OR7D2 | up |
| 4.614 | NM_017654 | SAMD9 | up |
| 4.603 | NM_030914 | C9orf74 | up |
| 4.591 | NM_003680 | YARS | up |
| 4.537 | NM_001005463 | EBF3 | up |
| 4.533 | NM_001402 | EEF1A1 | up |
| 4.505 | NM_001014291 | SPRR2G | up |
| 4.488 | NM_006761 | YWHAE | up |
| 4.471 | NM_152905 | NEDD1 | up |
| 4.459 | NM_213596 | FOXN4 | up |
| 4.432 | NM_198970 | AES | up |
| 4.423 | NM_024831 | NCOA6IP | up |
| 4.384 | NM_004431 | EPHA2 | up |
| 4.381 | NM_173197 | KCNIP2 | up |
| 4.359 | NM_005334 | HCFC1 | up |
| 4.357 | NM_002156 | HSPD1 | up |
| 4.319 | NM_004078 | CSRP1 | up |
| 4.29 | NM_014365 | HSPB8 | up |
| 4.289 | NM_001712 | CEACAM1 | up |
| 4.286 | NM_003012 | SFRP1 | up |
| 4.279 | NM_021149 | COTL1 | up |
| 4.272 | NM_006761 | YWHAE | up |
| 4.272 | NM_183422 | TSC22D1 | up |
| 4.262 | NM_003791 | MBTPS1 | up |
| 4.259 | NM_001202513 | MXD1 | up |
| 4.257 | NM_005159 | ACTC | up |
| 4.256 | NM_001030287 | ATF3 | up |
| 4.235 | NM_033375 | MYO1C | up |
| 4.232 | AK021593 | AK021593 | up |
| 4.219 | NM_002577 | PAK2 | up |
| 4.21 | NM_005505 | SCARB1 | up |
| 4.192 | NM_001008270 | LOC136242 | up |
| 4.191 | NM_006319 | CDIPT | up |
| 4.188 | NM_020350 | AGTRAP | up |
| 4.124 | NM_052892 | PKD1L2 | up |
| 4.101 | NM_003330 | TXNRD1 | up |
| 4.069 | NM_003133 | SRP9 | up |
| 4.053 | NM_004910 | PITPNM1 | up |
| 4.042 | NM_001520 | GTF3C1 | up |
| 4.035 | NM_006601 | PTGES3 | up |
| 4.028 | NM_203446 | SYNJ1 | up |
| 4.005 | NM_005345 | HSPA1A | up |
| 3.994 | NM_006815 | RNP24 | up |
| 3.981 | NM_052853 | ADCK2 | up |
| 3.98 | NM_015966 | C20orf47 | up |
| 3.967 | NM_007253 | CYP4F8 | up |
| 3.964 | NM_203364 | M11S1 | up |
| 3.944 | NM_006109 | SKB1 | up |
| 3.938 | NM_025207 | FLAD1 | up |
| 3.916 | NM_015313 | ARHGEF12 | up |
| 3.915 | NM_015383 | DJ328E19.C1.1 | up |
| 3.9 | NM_139239 | TA-NFKBH | up |
| 3.895 | NM_001013631 | HNRPCL1 | up |
| 3.895 | NM_007166 | PICALM | up |
| 3.873 | NM_153225 | RPESP | up |
| 3.866 | NM_033360 | KRAS | up |
| 3.863 | NM_004924 | ACTN4 | up |
| 3.818 | NM_001145770 | GPR56 | up |
| 3.816 | NM_006453 | TBL3 | up |
| 3.768 | NM_003040 | SLC4A2 | up |
| 3.717 | NM_030752 | TCP1 | up |
| 3.682 | NM_000528 | MAN2B1 | up |
| 3.677 | NM_002116 | HLA-A | up |
| 3.666 | NM_004418 | DUSP2 | up |
| 3.661 | NM_006412 | AGPAT2 | up |
| 3.656 | NM_007003 | PAGE4 | up |
| 3.654 | NM_020417 | TBX20 | up |
| 3.65 | NM_002164 | INDO | up |
| 3.635 | NM_000063 | C2 | up |
| 3.625 | NM_020441 | CORO1B | up |
| 3.607 | NM_006601 | PTGES3 | up |
| 3.599 | NM_015914 | TXNDC11 | up |
| 3.583 | NM_016284 | CNOT1 | up |
| 3.575 | NM_014445 | SERP1 | up |
| 3.567 | NM_133376 | ITGB1 | up |
| 3.547 | NM_031935 | HMCN1 | up |
| 3.536 | NM_006480 | RGS14 | up |
| 3.533 | NM_018708 | FEM1A | up |
| 3.529 | NM_017699 | SIDT1 | up |
| 3.522 | NM_001008392 | CTDSPL | up |
| 3.521 | NM_016143 | NSFL1C | up |
| 3.512 | NM_198584 | CA13 | up |
| 3.511 | NM_000550 | TYRP1 | up |
| 3.509 | NM_001008392 | CTDSPL | up |
| 3.504 | NM_006815 | RNP24 | up |
| 3.49 | NM_003440 | ZNF140 | up |
| 3.485 | NM_002480 | PPP1R12A | up |
| 3.481 | NM_002337 | LRPAP1 | up |
| 3.475 | NM_181526 | MYL9 | up |
| 3.472 | NM_001012505 | FOXP1 | up |
| 3.469 | NM_007111 | TFDP1 | up |
| 3.466 | NM_000651 | CR1 | up |
| 3.458 | NM_002205 | ITGA5 | up |
| 3.456 | NM_002301 | LDHC | up |
| 3.456 | NM_002166 | ID2 | up |
| 3.45 | NM_001017421 | FKSG30 | up |
| 3.447 | NM_006243 | PPP2R5A | up |
| 3.446 | NM_001005237 | OR51G1 | up |
| 3.445 | NM_002125 | HLA-DRB5 | up |
| 3.441 | NM_001101 | ACTB | up |
| 3.427 | NM_007332 | TRPA1 | up |
| 3.417 | NM_014400 | C4.4A | up |
| 3.411 | NM_012100 | DNPEP | up |
| 3.408 | NM_173496 | MPP7 | up |
| 3.401 | NM_032439 | PHYHIPL | up |
| 3.396 | NM_002070 | GNAI2 | up |
| 3.396 | NM_052847 | GNG7 | up |
| 3.395 | AK093664 | RYR3 | up |
| 3.394 | NM_174958 | ATP2A3 | up |
| 3.394 | NM_017910 | FLJ20628 | up |
| 3.374 | NM_006129 | BMP1 | up |
| 3.372 | NM_018229 | C14orf108 | up |
| 3.368 | NM_033430 | PDE5A | up |
| 3.363 | NM_001456 | FLNA | up |
| 3.358 | NM_015556 | SIPA1L1 | up |
| 3.346 | NM_147198 | WFDC9 | up |
| 3.339 | NM_003220 | TFAP2A | up |
| 3.338 | NM_032264 | AE2 | up |
| 3.332 | NM_020145 | SH3GLB2 | up |
| 3.321 | NM_080392 | PTP4A2 | up |
| 3.318 | NM_005684 | GPR52 | up |
| 3.318 | NM_016279 | CDH9 | up |
| 3.318 | NM_016522 | HNT | up |
| 3.314 | NM_001386 | DPYSL2 | up |
| 3.314 | NM_001005354 | DKFZp564J157 | up |
| 3.311 | NM_177951 | PPM1A | up |
| 3.307 | NM_134268 | CYGB | up |
| 3.305 | NM_006941 | SOX10 | up |
| 3.301 | NM_019082 | DDX56 | up |
| 3.298 | NM_004428 | EFNA1 | up |
| 3.296 | NM_006185 | NUMA1 | up |
| 3.296 | NM_001312 | CRIP2 | up |
| 3.294 | NM_024069 | MGC2749 | up |
| 3.274 | NM_013374 | PDCD6IP | up |
| 3.271 | NM_005993 | TBCD | up |
| 3.269 | NM_020831 | MKL1 | up |
| 3.267 | NM_032638 | GATA2 | up |
| 3.258 | NM_001317 | CSH1 | up |
| 3.254 | NM_014604 | TAX1BP3 | up |
| 3.253 | NM_005672 | PSCA | up |
| 3.253 | NM_015251 | ASCIZ | up |
| 3.249 | NM_002383 | MAZ | up |
| 3.246 | NM_012089 | ABCB10 | up |
| 3.244 | NM_001038 | SCNN1A | up |
| 3.242 | NM_174947 | C19orf30 | up |
| 3.239 | NM_006761 | YWHAE | up |
| 3.236 | NM_032389 | ZNF289 | up |
| 3.231 | NM_005080 | XBP1 | up |
| 3.225 | NM_012305 | AP2A2 | up |
| 3.224 | NM_177998 | OTOP1 | up |
| 3.218 | NM_019606 | FLJ20257 | up |
| 3.218 | NM_016559 | PEX5L | up |
| 3.216 | NM_170697 | ALDH1A2 | up |
| 3.213 | NM_022112 | P53AIP1 | up |
| 3.21 | NM_001681 | ATP2A2 | up |
| 3.202 | NM_005437 | NCOA4 | up |
| 3.202 | NM_025147 | FLJ13448 | up |
| 3.197 | NM_000478 | ALPL | up |
| 3.189 | NM_172345 | SPAG9 | up |
|  |  |  |  |
| 9.500 | NM_006037 | HDAC4 | down |
| 9.620 | AK124869 | LOC400745 | down |
| 9.499 | NM_006898 | HOXD3 | down |
| 8.783 | NM_015308 | FNBP4 | down |
| 8.733 | NM_152424 | FLJ39827 | down |
| 8.623 | NM_152748 | KIAA1324L | down |
| 8.583 | NM_033305 | VPS13A | down |
| 8.482 | NM_025114 | Cep290 | down |
| 8.472 | NM_000975 | RPL11 | down |
| 8.353 | NM_021211 | LOC58486 | down |
| 8.321 | NM_001077511 | TCF19 | down |
| 8.256 | NM_006202 | PDE4A | down |
| 8.216 | NM_015679 | TRUB2 | down |
| 8.116 | NM_001812 | CENPC1 | down |
| 7.913 | NM_007053 | CD160 | down |
| 7.943 | NM_001039802 | CDC42 | down |
| 7.972 | NM_133457 | EMID2 | down |
| 7.990 | NM_033305 | VPS13A | down |
| 7.972 | NM_022482 | ZNF336 | down |
| 7.943 | NM_133457 | EMID2 | down |
| 7.913 | NM_000637 | GSR | down |
| 7.913 | NM_015442 | CNOT10 | down |
| 7.899 | NM_015969 | MRPS17 | down |
| 7.886 | NM_002015 | FOXO1A | down |
| 7.858 | NM_024077 | SECISBP2 | down |
| 7.851 | NM_001554 | CYR61 | down |
| 7.845 | NM_001969 | EIF5 | down |
| 7.820 | NM_000584 | IL-8 | down |
| 7.759 | NM_006836 | GCN1L1 | down |
| 7.752 | NM_033306 | CASP4 | down |
| 7.713 | NM_015982 | YBX2 | down |
| 7.621 | NM_000658 | AIRE | down |
| 7.541 | NM_033194 | HSPB9 | down |
| 7.541 | NM_013363 | PCOLCE2 | down |
| 7.516 | NM_018667 | SMPD3 | down |
| 7.485 | NM_173466 | DKFZp434P055 | down |
| 7.418 | NM_001111307 | PDE4A | down |
| 7.413 | NM_000526 | KRT14 | down |
| 7.387 | NM_001911 | CTSG | down |
| 7.352 | NM_172037 | RDH10 | down |
| 7.334 | NM_006744 | RBP4 | down |
| 7.291 | NM_015421 | C16orf51 | down |
| 7.251 | NM_001011546 | DSTN | down |
| 7.122 | NM_006792 | MORF4 | down |
| 6.908 | NM_001645 | APOC1 | down |
| 6.771 | NM_175709 | CBX7 | down |
| 6.448 | NM_032378 | EEF1D | down |
| 6.439 | NM_017458 | MVP | down |
| 6.423 | NM_015442 | CNOT10 | down |
| 6.401 | NM_005214 | CTLA4 | down |
| 6.272 | NM_021008 | DEAF1 | down |
| 6.203 | NM_003292 | TPR | down |
| 6.150 | NM_018281 | ECHDC2 | down |
| 6.122 | NM_015310 | PSD3 | down |
| 6.088 | NM_203448 | MGC21881 | down |
| 6.077 | NM_001017922 | ERMAP | down |
| 5.961 | NM_006561 | CUGBP2 | down |
| 5.980 | NM_007053 | CD160 | down |
| 5.961 | NM_000091 | COL4A3 | down |
| 5.863 | NM_006714 | SMPDL3A | down |
| 5.861 | NM_020179 | FN5 | down |
| 5.856 | NM_022371 | TOR3A | down |
| 5.755 | NM_004239 | TRIP11 | down |
| 5.753 | NM_015208 | ANKRD12 | down |
| 5.743 | NM_032265 | ZMYND15 | down |
| 5.731 | NM_021821 | MRPS35 | down |
| 5.710 | NM_018719 | CDCA7L | down |
| 5.524 | AL834432 | CCNL2 | down |
| 5.519 | NM_017745 | BCOR | down |
| 5.410 | NM_000565 | IL6R | down |
| 5.309 | NM_002599 | PDE2A | down |
| 5.297 | NM_000715 | C4BPA | down |
| 5.292 | NM_000846 | GSTA2 | down |
| 5.263 | NM_003629 | PIK3R3 | down |
| 5.181 | NM_001165 | BIRC3 | down |
| 5.138 | NM_001130964 | PLCD1 | down |
| 5.060 | NM_005557 | KRT16 | down |
| 4.989 | NM_001333 | CTSL2 | down |
| 4.964 | NM_001252641 | URI1 | down |
| 4.961 | NM_153211 | C18orf17 | down |
| 4.882 | NM_052828 | TRIM10 | down |
| 4.854 | NM_003064 | SLPI | down |
| 4.817 | NM_001080855 | PXN | down |
| 4.799 | NM_015378 | VPS13D | down |
| 4.783 | NM_015600 | C20orf22 | down |
| 4.773 | NM_002700 | POU4F3 | down |
| 4.764 | NM_001003395 | TPD52L1 | down |
| 4.731 | NM_015679 | TRUB2 | down |
| 4.702 | NM_002648 | PIM1 | down |
| 4.689 | NM_000715 | C4BPA | down |
| 4.684 | NM_001042544 | LTBP4 | down |
| 4.651 | NM_033342 | TRIM7 | down |
| 4.639 | NM_006186 | NR4A2 | down |
| 4.590 | NM_006468 | POLR3C | down |
| 4.574 | NM_175736 | FMNL3 | down |
| 4.458 | NM_001007527 | LMBRD2 | down |
| 4.439 | NM_005613 | RGS4 | down |
| 4.437 | NM_005902 | SMAD3 | down |
| 4.417 | NM_020990 | CKMT1B | down |
| 4.392 | NM_138399 | TMEM44 | down |
| 4.358 | NM_019101 | APOM | down |
| 4.334 | NM_001671 | ASGR1 | down |
| 4.325 | NM_032682 | FOXP1 | down |
| 4.282 | NM_000637 | GSR | down |
| 4.200 | NM_014948 | UBOX5 | down |
| 4.112 | NM_002078 | GOLGA4 | down |
| 3.992 | NM_033402 | KIAA1764 | down |
| 3.987 | NM_021948 | BCAN | down |
| 3.970 | NM_198278 | NPNT | down |
| 3.946 | NM_016652 | CRNKL1 | down |
| 3.940 | NM_002470 | MYH3 | down |
| 3.929 | NM_006037 | HDAC4 | down |
| 3.921 | NM_014797 | ZBTB24 | down |
| 3.916 | NM_000343 | SLC5A1 | down |
| 3.907 | NM_032047 | B3GNT5 | down |
| 3.870 | NM_173491 | LSM11 | down |
| 3.863 | NM_001271998 | RAB4A | down |
| 3.840 | NM_031283 | TCF7L1 | down |
| 3.818 | NM_172193 | KLHDC1 | down |
| 3.772 | NM_006673 | ARID5A | down |
| 3.754 | NM_052819 | CARD14 | down |
| 3.751 | NM_015124 | DIP | down |
| 3.739 | NM_015710 | GLTSCR2 | down |
| 3.729 | NM_012331 | MSRA | down |
| 3.716 | NM_001417 | EIF4B | down |
| 3.706 | NM_023075 | MPPE1 | down |
| 3.681 | NM_013231 | FLRT2 | down |
| 3.666 | NM_005373 | MPL | down |
| 3.648 | NM_016089 | ZNF589 | down |
| 3.630 | NM_194441 | BTN3A1 | down |
| 3.588 | NM_033292 | CASP1 | down |
| 3.537 | NM_018288 | PHF10 | down |
| 3.431 | NM_006938 | SNRPD1 | down |
| 3.411 | NM_032795 | RPUSD4 | down |
| 3.378 | NM_015370 | HS747E2A | down |
