# Supplementary material for: VEZT, a Novel Putative Tumor Suppressor, Suppresses the Growth and Tumorigenicity of Gastric Cancer
Source: PLoS One. 2013 Sep 17;8(9):e74409. doi: 10.1371/journal.pone.0074409 (PMC3775783; doi:10.1371/journal.pone.0074409)
Supplement: Table S4 — 23 Upregulated and 26 downregulated target genes were identified by global microarray analysis. (DOC) [file pone.0074409.s005.doc]

| Target gene identification by global microarray analysis | | | | |
| --- | --- | --- | --- | --- |
| **Number** | **Fold Change** | **Genbank** | **GeneSymbol** | **Regulation** |
| 1 | 3.472 | NM_001012505 | FOXP1 | up |
| 2 | 3.445 | NM_002125 | HLA-DRB5 | up |
| 3 | 3.717 | NM_030752 | TCP1 | up |
| 4 | 4.191 | NM_006319 | CDIPT | up |
| 5 | 14.870 | NM_001199917 | PGM3 | up |
| 6 | 7.484 | NM_003011 | SET | up |
| 7 | 3.458 | NM_002205 | ITGA5 | up |
| 8 | 4.357 | NM_002156 | HSPD1 | up |
| 9 | 3.509 | NM_001008392 | CTDSPL | up |
| 10 | 3.866 | NM_033360 | KRAS | up |
| 11 | 4.256 | NM_001030287 | ATF3 | up |
| 12 | 3.575 | NM_014445 | SERP1 | up |
| 13 | 3.567 | NM_133376 | ITGB1 | up |
| 14 | 6.358 | NM_030763 | HMGN5 | up |
| 15 | 4.533 | NM_001402 | EEF1A1 | up |
| 16 | 3.818 | NM_001145770 | GPR56 | up |
| 17 | 4.259 | NM_001202513 | MXD1 | up |
| 18 | 3.298 | NM_004428 | EFNA1 | up |
| 19 | 4.537 | NM_001005463 | EBF3 | up |
| 20 | 6.527 | NM_003819 | PABPC4 | up |
| 21 | 3.511 | NM_000550 | TYRP1 | up |
| 22 | 6.211 | NM_015914 | TXNDC11 | up |
| 23 | 5.033 | NM_003801 | GPAA1 | up |
| 1 | 3.929 | NM_006037 | HDAC4 | down |
| 2 | 3.706 | NM_023075 | MPPE1 | down |
| 3 | 5.731 | NM_021821 | MRPS35 | down |
| 4 | 4.817 | NM_001080855 | PXN | down |
| 5 | 3.863 | NM_001271998 | RAB4A | down |
| 6 | 5.138 | NM_001130964 | PLCD1 | down |
| 7 | 4.689 | NM_000715 | C4BPA | down |
| 8 | 9.499 | NM_006898 | HOXD3 | down |
| 9 | 7.541 | NM_033194 | HSPB9 | down |
| 10 | 7.418 | NM_001111307 | PDE4A | down |
| 11 | 4.731 | NM_015679 | TRUB2 | down |
| 12 | 7.251 | NM_001011546 | DSTN | down |
| 13 | 5.980 | NM_007053 | CD160 | down |
| 14 | 6.908 | NM_001645 | APOC1 | down |
| 15 | 7.851 | NM_001554 | CYR61 | down |
| 16 | 7.943 | NM_133457 | EMID2 | down |
| 17 | 8.583 | NM_033305 | VPS13A | down |
| 18 | 7.820 | NM_000584 | IL-8 | down |
| 19 | 6.122 | NM_015310 | PSD3 | down |
| 20 | 4.282 | NM_000637 | GSR | down |
| 21 | 6.423 | NM_015442 | CNOT10 | down |
| 22 | 4.964 | NM_001252641 | URI1 | down |
| 23 | 4.684 | NM_001042544 | LTBP4 | down |
| 24 | 8.321 | NM_001077511 | TCF19 | down |
| 25 | 7.943 | NM_001039802 | CDC42 | down |
| 26 | 7.972 | NM_022482 | ZNF336 | down |
