# Supplementary material for: VEZT, a Novel Putative Tumor Suppressor, Suppresses the Growth and Tumorigenicity of Gastric Cancer
Source: PLoS One. 2013 Sep 17;8(9):e74409. doi: 10.1371/journal.pone.0074409 (PMC3775783; doi:10.1371/journal.pone.0074409)
Supplement: Table S5 — Verified genes and their functions by real-time PCR. We examined 8 Upregulated and 10 downregulated target genes by real-time PCR and listed their funtions. (DOC) [file pone.0074409.s006.doc]

| Verified genes and their functions by real-time PCR | | | | |  |
| --- | --- | --- | --- | --- | --- |
| **Genes** | **Accession number** | **Regulation** | **Fold change** | **Functions** |  |
| HMGN5 | NM_030763 | up | 6.358 | a nucleosomal binding and transcriptional  activating protein;inhibits the growth and invasion |  |
| ITGA5 | NM_002205 | up | 3.458 | the integrin alpha chain family;adhesion |  |
| PGM3 | NM_001199917 | up | 14.870 | a member of the phosphohexose mutase family;  promoted cell death in LNCaP prostate cancer cells |  |
| MXD1 | NM_001202513 | up | 4.259 | a member of the MYC/MAX/MAD network of basic  helix-loop-helix leucine zipper transcription  factors;inhibit migration and invasion |  |
| ATF3 | NM_001030287 | up | 4.256 | a member of the mammalian activation transcription  factor/cAMP responsive element-binding (CREB)  proteinfamily of transcription factors;Transcription regulators |  |
| CDIPT | NM_006319 | up | 4.191 | cell growth, calcium metabolism, and protein  kinase C activity |  |
| FOXP1 | NM_001012505 | up | 3.472 | subfamily P of the forkhead box (FOX) transcription factor family;This gene may act as a tumor suppressor |  |
| GPR56 | NM_001145770 | up | 3.818 | a member of the G protein-coupled receptor family; overexpression of this gene can suppress tumor growth and metastasis |  |
| URI1 | NM_001252641 | down | 4.964 | member of the prefoldin family of molecular chaperones; promoting cell survival |  |
| HOXD3 | NM_006898 | down | 9.499 | the homeobox family of genes ;the regulation of cell adhesion processes |  |
| PLCD1 | NM_001130964 | down | 5.138 | a member of the phospholipase C family;Cell-adhesion molecules |  |
| RAB4A | NM_001271998 | down | 3.863 | a member of the largest group in the Ras superfamily of small GTPases;regulating the recycling of receptors from endosomes to the plasma membrane |  |
| PXN | NM_001080855 | down | 4.817 | a cytoskeletal protein involved in actin-membrane attachment at sites of cell adhesion to the extracellular matrix;cell motility |  |
| LTBP4 | NM_001042544 | down | 4.684 | this gene binds transforming growth factor beta (TGFB) as it is secreted and targeted to the extracellular matrix;regulation of cell growth |  |
| TCF19 | NM_001077511 | down | 8.321 | regulation of transcription from RNA polymerase II promoter ;intracellular signal transduction;cell cycle |  |
| IL-8 | NM_000584 | down | 7.820 | this gene is a member of the CXC chemokine family; Chemokine and chemokine receptors |  |
| CDC42 | NM_001039802 | down | 7.943 | regulates signaling pathways that control diverse cellular functions including cell morphology, migration, endocytosis and cell cycle progression; promote migration and invasion |  |
| DSTN | NM_001011546 | down | 7.251 | the actin-binding proteins ADF family;promote  migration and invasion |  |
